# Traumatic Brain Injury-Induced Sex-Dependent Changes in Late-Onset Sensory Hypersensitivity and Glutamate Neurotransmission

**DOI:** 10.3389/fneur.2020.00749

**Published:** 2020-08-05

**Authors:** Gokul Krishna, Caitlin Bromberg, Emily Charlotte Connell, Erum Mian, Chengcheng Hu, Jonathan Lifshitz, P. David Adelson, Theresa Currier Thomas

**Affiliations:** ^1^Department of Child Health, University of Arizona College of Medicine – Phoenix, Phoenix, AZ, United States; ^2^Barrow Neurological Institute at Phoenix Children's Hospital, Phoenix, AZ, United States; ^3^Department of Biology and Biochemistry, University of Bath, Bath, United Kingdom; ^4^Department of Epidemiology and Biostatistics, University of Arizona, Tucson, AZ, United States; ^5^Phoenix VA Health Care System, Phoenix, AZ, United States

**Keywords:** traumatic brain injury, sex difference, glutamate, behavior, neurotransmitters, whisker, microelectrode arrays, estrous

## Abstract

Women approximate one-third of the annual 2.8 million people in the United States who sustain traumatic brain injury (TBI). Several clinical reports support or refute that menstrual cycle-dependent fluctuations in sex hormones are associated with severity of persisting post-TBI symptoms. Previously, we reported late-onset sensory hypersensitivity to whisker stimulation that corresponded with changes in glutamate neurotransmission at 1-month following diffuse TBI in male rats. Here, we incorporated intact age-matched naturally cycling females into the experimental design while monitoring daily estrous cycle. We hypothesized that sex would not influence late-onset sensory hypersensitivity and associated *in vivo* amperometric extracellular recordings of glutamate neurotransmission within the behaviorally relevant thalamocortical circuit. At 28 days following midline fluid percussion injury (FPI) or sham surgery, young adult Sprague-Dawley rats were tested for hypersensitivity to whisker stimulation using the whisker nuisance task (WNT). As predicted, both male and female rats showed significantly increased sensory hypersensitivity to whisker stimulation after FPI, with females having an overall decrease in whisker nuisance scores (sex effect), but no injury and sex interaction. In males, FPI increased potassium chloride (KCl)-evoked glutamate overflow in primary somatosensory barrel cortex (S1BF) and ventral posteromedial nucleus of the thalamus (VPM), while in females the FPI effect was discernible only within the VPM. Similar to our previous report, we found the glutamate clearance parameters were not influenced by FPI, while a sex-specific effect was evident with female rats showing a lower uptake rate constant both in S1BF and VPM and longer clearance time (in S1BF) in comparison to male rats. Fluctuations in estrous cycle were evident among brain-injured females with longer diestrus (low circulating hormone) phase of the cycle over 28 days post-TBI. Together, these findings add to growing evidence indicating both similarities and differences between sexes in a chronic response to TBI. A better understanding of the influence of gonadal hormones on behavior, neurotransmission, secondary injury and repair processes after TBI is needed both clinically and translationally, with potential impact on acute treatment, rehabilitation, and symptom management.

## Highlights

- Diffuse traumatic brain injury (TBI)-induces sensory hypersensitivity in males and females.
- TBI increased evoked glutamate overflow in S1BF and VPM of males and VPM of females.
- Females displayed sex-specific changes in glutamate in comparison to males.
- TBI alters estrous cyclicity with prolonged low hormonal diestrus over 28 days post-injury.

## Introduction

The global burden of traumatic brain injury (TBI) is estimated at 69 million cases each year (1). Mild TBI makes up 75% of all reported TBIs, commonly associated with falls, assault, sports, and military activities (2). After mild TBI, patients are at higher risk for developing long-lasting neurological morbidities that have detrimental effects on functional ability (3). Persisting post-TBI symptoms include headaches, cognitive disabilities (4), sensory deficits (5), sexual dysfunction (6), mood and anxiety disorders (7, 8), and increases the risk for second brain injuries (9). Specifically, symptoms associated with cognition (memory, executive function, and processing speed), emotional processing (mood and social), and sensory perception (vision and auditory) (10) can hamper recovery. Reports of complaints associated with post-TBI symptoms vary widely in mild TBI literature (11, 12). In recent publications from the UPFRONT studies, 85–90% of mild TBI patients had complaints at 2-weeks post-injury (13, 14). When evaluating the 10% of patients that did not have complaints at 2-weeks post-injury, over 50% developed complaints over the following 12 months (14). Overall, more complaints were in mild TBI patients with a psychiatric history with more complaints in females compared to males. The De Koning study highlights the prevalence for late-onset symptoms, for which few preclinical models exist.

One-third of TBI patients each year in the United States are women, where reports support or refute whether sex differences influence severity and duration of chronic post-TBI symptoms, with a greater amount of data supporting sex differences (15–18). In particular, a largely overlooked population are victims of domestic violence who are predominantly women and children (19). Reports indicate that women of reproductive age are more likely than men to report severe persisting post-TBI symptoms (5, 20) and data suggest that they sustain significantly higher TBI rates (21). The number of women patients receiving health care from Veterans Affairs medical centers has also substantially increased over the past years (22). Moreover, clinical observations suggest many female TBI survivors experience menstrual problems such as amenorrhea, irregular cycles, infertility, and postpartum complications (23–25). Among the few known physiological factors, fluctuating sex hormone levels have been indicated in the disparity of chronic symptoms associated with persisting post-TBI symptoms in females, with injury during the luteal phase of the menstrual cycle associated with worse outcomes (26). Clinically and translationally, TBI-induced chronic ovarian hormonal deficiencies have been shown to contribute to behavioral deficits (27, 28). Further, TBI prolongs the low circulating ovarian hormone phase that is associated with cognitive and sensorimotor deficits in rodents (29). Since brain injury is characterized by a wide heterogeneity in pathophysiological mechanisms, evaluating for variability across the sexes may be crucial for patient stratification and treatment as well as translationally relevant study designs.

Mild TBI initiates shearing forces leading to diffuse axonal injury (DAI), synaptic deafferentation, vascular permeability, and inflammation that progress toward dysfunction of neural circuitry that can be replicated, in part, in experimental models of TBI using midline fluid percussion injury (FPI) (30). DAI initiates a long-term process that elicits both degenerative and regenerative (dendritic and synaptic sprouting) responses (31). Abnormal pathophysiology drives maladaptive compensation in neurotransmitter systems of long-range projections relevant to post-TBI symptoms [reviewed in (32)]. The somatosensory thalamocortical projections of the whisker barrel circuit (WBC) in rodents are essential components for sensory processing in rats (33). Within this circuitry, glutamatergic input from the principal trigeminal nucleus (PrV) projects to the contralateral ventral posteromedial nucleus of the thalamus (VPM) that extends long range glutamatergic projections to the primary somatosensory cortex (S1BF). Midline FPI causes pathological alterations along the whisker circuit that are characterized by changes in glutamate neurotransmission, axotomy, dendritic atrophy, circuit reorganization, and gliosis that parallel the development of late-onset sensory hypersensitivity in male rats after TBI (34–38), similar to agitation presented in human TBI (39). At 1-week post-injury, rats exposed to FPI did not exhibit sensitivity to whisker stimulation (40), similar to clinical reports in the De Koning study, where symptoms developed over time (14).

One elegant approach to studying the circuit function is taking advantage of biosensor technology to assess neurotransmission responsible for the development of chronic morbidities after TBI (41). Our previous work in male brain-injured animals demonstrated hypersensitive glutamate signaling associated with the severity of late-onset hypersensitivity to whisker stimulation as a consequence of pre-synaptic glutamate release (40). Here, we replicate previous studies in males with the inclusion of systematic estrous cycle assessment in naturally cycling female rats to determine if sex plays an intrinsic role in TBI-induced sensory hypersensitivity associated with changes in glutamate neurotransmission.

## Materials and Methods

### Chemicals and Reagents

1,3 phenylenediamine (mPD 99%, cat. no. 78450, Acros Organics, NJ), bovine serum albumin (BSA, cat. no. A3059), glutaraldehyde, L-ascorbic acid (≥99%, cat. no. A5960), L-glutamic acid (≥99%, HPLC grade, cat. no. G1626) and L-Glutamate oxidase from *Streptomyces* sp. with rated activity of ≥10 U mg^−1^ (Lowry's method) (cat. no. G59211UN) were purchased from Millipore Sigma. Phosphate-buffered saline (PBS, pH 7.4) was composed of sodium phosphate dibasic (Na_2_HPO_4_, cat. no. BP332), sodium phosphate (NaHPO HO, cat. no. BP330), and sodium chloride (NaCl, cat. no. S271). Ultrapure water, generated using a Millipore Milli-Q Water Purification System, was used for preparation of all solutions used in this work.

### Animals

Young adult male and naturally cycling female 3–4 month old Sprague-Dawley rats (330–350 g and 210–230 g, respectively, at the time of surgery; Envigo, Indianapolis, IN) were same-sex pair housed (2 animals/cage) and allowed 1-week of acclimation to a normal 12 h light/dark cycle with access to food (Teklad 2918, Envigo, Indianapolis, IN) and water *ad libitum*. All study protocols were approved by the Institutional Animal Care and Use Committee, University of Arizona College of Medicine—Phoenix (Protocol No. 18-384) and were conducted in adherence to guidelines established by the National Institutes of Health (NIH) Guidelines for the Care and Use of Laboratory Animals. Based on our previous reports in males (34, 40), group sizes of 14 rats are sufficient to detect a three-point change in whisker nuisance scores between groups with 90% power (2-tailed; μ1 = 3.8, μ2 = 6.8, σ = 2.5; significance level *P* = 0.05).

### Midline Fluid Percussion Injury (FPI)

#### Surgical Procedure

Following acclimation to the vivarium for 1 week, all rats were habituated to human handling for 5 days prior to surgery. Cages of rats were randomized into either injured (midline FPI) or sham surgery groups. We followed our standard midline FPI surgery protocol as previously described (42). In brief, rat surgeries were performed under isoflurane anesthesia (5% induction and 2.5% maintenance vaporized in 100% O_2_ with flow rate of 0.8 L/min via nosecone) while being secured on a stereotaxic frame (Kopf Instruments, Tujunga, CA) and absent toe pinch response. The body temperature was maintained at 37°C throughout the surgical period using a thermostatic heating pad. Ophthalmic ointment was applied to the eyes to prevent drying (07-888-2572, Patterson Veterinary, CO). The scalp was shaved and cleaned with alternating betadine and ethanol scrubs. A 4.8 mm diameter circular craniectomy was centered on the sagittal suture midway between bregma and lambda using a trephine, ensuring that the underlying dura, and superior sagittal sinus were not damaged. An injury hub, using the female portion of a 20-gauge Luer-Lock needle hub (cut and beveled), was placed directly above and in-line with the craniectomy site. A stainless-steel anchoring screw was then placed in the right frontal bone using a hand-drill. The injury hub was affixed over the craniectomy using cyanoacrylate gel and dental cement (Hygenic Corp., Akron, OH). After the dental cement hardened, the hub was filled with 0.9% sterile saline. The incision was then partially sutured closed on both the anterior and posterior edges with 4.0 Ethilon suture (Med-Vet International, Mettawa, IL) topical lidocaine and antibiotic ointment were applied. Rats were returned to a warmed holding cage and monitored until ambulatory (~60–90 min).

#### Injury Induction

After cranial surgery, rats were allowed to recover for ~2 h in the recovery chamber during which they were observed for ambulatory movement and any signs of ill health. After ensuring successful surgery, rats were re-anesthetized using 5% isoflurane in 100% oxygen for 3 min. The injury hub was filled with 0.9% sterile saline and attached to the fluid percussion device (Custom Design and Fabrication, Virginia Commonwealth University, Richmond, VA). At the return of the toe pinch withdrawal reflex, a moderate fluid pulse [(in atm): Males, 1.9–2.1 and Females, 1.8–2.0] was administered by releasing the pendulum (16.5 degrees for males and 16 degrees for females) onto the fluid-filled cylinder. Immediately after administration of the injury, the hub was removed *en bloc*, rats placed on the heated recovery chamber, and monitored for presence of apnea time, fencing response and return of righting reflex. The rats were then re-anesthetized to inspect for signs of hematoma and herniation at the site of injury. The surgical wound was cleaned, stapled closed, and topical lidocaine and antibiotic ointment were applied at the surgical site. Inclusion criteria required that injured rats have a righting reflex time ranging from 5 to 9 min and display a fencing response (43). After regaining the righting reflex, rats were placed in a holding cage for ~1 h until regaining normal ambulatory behavior before being returned to standard vivarium conditions. Sham animals underwent identical procedures without dropping the pendulum to induce the injury. Adequate post-operative care was administered for 3–5 days after surgery where all the animals were assessed for body weight changes, physical examination, suture site, and pain or distress using a standardized protocol. Rats that lost more than 20% body weight or scored poorly during this post-operative time were euthanized (<10%). All rats included in this study had (i) same-sex cagemate, (ii) same-injury cagemate, (iii) a righting reflex between 5 and 9 min (FPI only), and (iv) a visible fencing response (FPI only).

### Estrous Cycle Analysis

**Figure 1:**
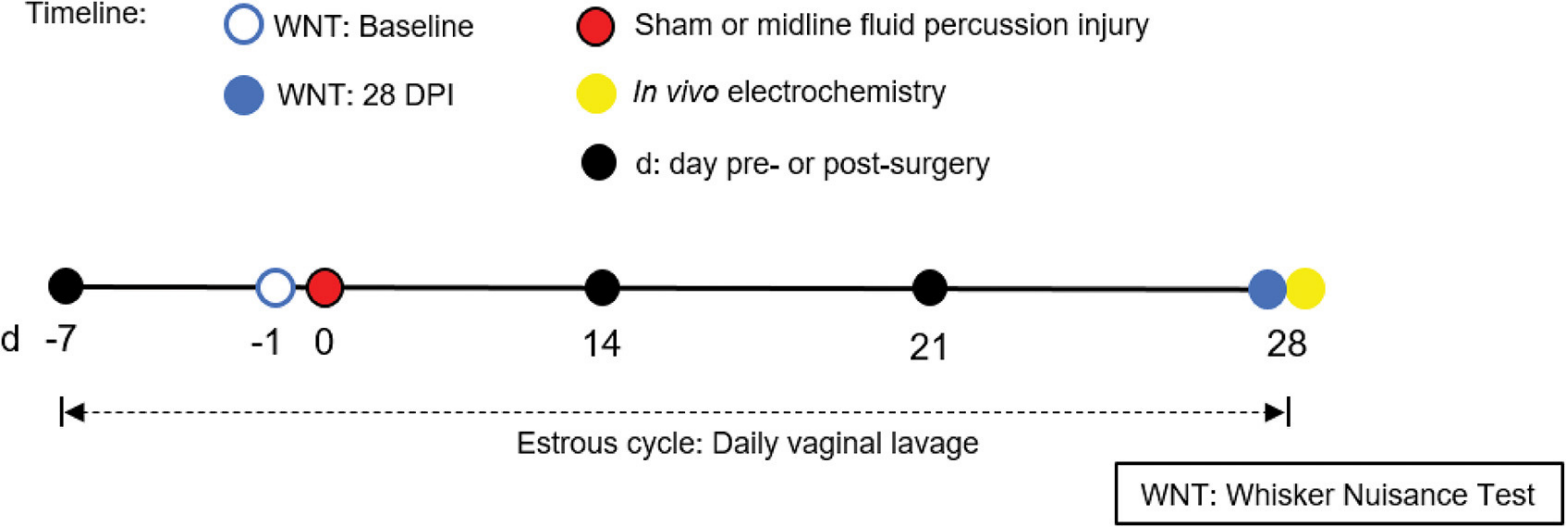
Timeline representing experimental design. Following acclimatization, young adult male and naturally cycling intact female Sprague-Dawley rats were habituated to handling. In females, estrous cycle was tracked daily, from 7 days pre-injury to 28 days post-injury. At day -1 (day before surgery), sham or midline FPI rats were tested with a baseline whisker nuisance task (WNT), where rats scoring >3 were excluded from the experiment. Then, cages of age-matched male and female rats were randomized to either sham surgery or midline fluid percussion injury (FPI). The WNT was performed again on day 28 post-injury. Rats were anesthetized 30 min following the final behavioral testing for *in vivo* amperometric recordings. Immediately following recordings, animals were decapitated and brains were cryosectioned to confirm microelectrode array (MEA) placement.

Estrous cycle was tracked using daily vaginal lavage with sterile saline beginning on the day of handling (7 days prior to surgery) and ending at 28 days post-injury (DPI) to determine the estrous cyclicity (Figure 1). Cycle tracking was done by an investigator blinded to injury status. Three hundred microliter of sterile saline was gently pipetted into and out of the vagina and smeared onto a glass microscope slide. Estrous stage was immediately determined under the microscope (Nikon, 77007) fitted with a 20× objective and classified according to previously reported methods (44). Briefly, the metestrus was characterized by the appearance of small leukocytes with equally round nucleated epithelial cells, whereas, the diestrus had fewer leukocytes along with cornified epithelia. The smears from proestrus rats had predominantly round nucleated cells. In the estrus phase, non-nucleated cornified epithelial cells were predominant. Estrous cycles were tracked daily, between 07:00 a.m. and 12:00 p.m. to minimize variability due to diurnal variations. Male rats were habituated to handling daily. Given that on a daily basis female rats normally fall in one of the different stages of the estrous cycle, all female rats underwent either sham or midline FPI (as described above) regardless of their estrous phase.

### Behavioral Testing: Whisker Nuisance Task (WNT)

Behavioral response to whisker stimulation was assessed by whisker nuisance task (WNT), as described previously (34) at baseline (on the day before surgery) and 28 days post-injury by an investigator blinded to injury status. The test identifies sensory hypersensitivity when whiskers are manually stimulated in an open field (45). Briefly, each rat was individually placed in the middle of an open arena (16.5 × 38.1 × 60 cm, 7,362-cm^2^) and permitted free exploration of the chamber during which their whiskers of both mystacial pads were manually stimulated with a wooden applicator stick for 15 min with three consecutive 5-min periods and less than ≤ 60 s non-stimulation break between each period. For each 5 min period, the behavioral responses including movement, stance and body position, breathing, whisker position, whisking response, evading stimulation, response to stick presentation and grooming were assigned subjective behavioral scores on 0–2 point non-parametric scales. White noise of 70 dB was present at the testing times to mask external noise. *Data collection:* The primary method for data collection of behavioral phenotypes was evaluation and scoring using the published whisker nuisance task scoring sheet (34). A score of (0) indicated the rat showed *no response or “absent”* indicating normal response; (1) denoted the rat showed a *minor degree of expression or “present”*; and (2) the rat showed major *degree of expression or “profound”* exemplified with above behaviors indicating abnormal responses. The total scores were averaged across three 5-min stimulation bouts. The maximum whisker nuisance score was 16, with higher scores indicating multiple abnormal behavioral responses. From their homecages, rats were placed individually into another transport cage and moved to the separate behavioral testing room located within the same Laboratory Animal Care Facility and then returned to their home cages at the end of testing. The experimenter made minimal movements and no noise when collecting the behavioral data and used a timer to determine the testing period. To avoid the possibility of different experience or stressor condition, all rats spent brief, consistent time outside their homecages, during transport and in the behavioral testing suite to ensure they underwent similar experiences. Testing was conducted at the same time of day by the same observer, who was blinded to injury status. Animals were excluded if they had high (>3) basal whisker nuisance scores tested on the day before surgery.

### Electrochemistry: Enzyme-Based Microelectrode Arrays

Ceramic-based microelectrode arrays (MEAs) encompassing four platinum (Pt) recording surfaces (15 μm × 333 μm) aligned in a dual, paired configuration were prepared to measure glutamate for *in vivo* anesthetized recordings (S2 configuration; Quanteon, Nicholasville, KY). MEAs were fabricated for sensitive and selective measurements of glutamate as previously described (42, 46). The MEAs use glutamate oxidase (GluOx) to catalyze the oxidative deamination of glutamate leading to formation of hydrogen peroxide (H_2_O_2_) as a by-product. Electro-oxidation (at a potential of +0.7 V) of enzymatically generated H_2_O_2_ at the surface of the electrode generated the current output which was recorded and converted into glutamate concentration via an *in vitro* calibration factor. Further, interference from other electroactive neurotransmitters was excluded from the amperometric recordings by application of mPD coating to the electrode sites (40).

### MEA Modification for Glutamate Detection

Briefly, a solution of GluOx, bovine serum albumin (BSA), and glutaraldehyde (GA) was immobilized by chemical cross-linking onto two of the Pt electrode recording sites, enabling these sites to selectively detect glutamate levels with low limits of detection (42). The two sentinel channels were coated with only BSA and glutaraldehyde, recording everything except for glutamate (47). Enzyme immobilization was accomplished by chemical cross-linking using a solution of GluOx (400 U/ml), BSA (6 mg/ml), and GA (0.075%). A needle attached to a 10 μL Hamilton syringe tip was used to coat MEAs under a stereomicroscope with a ~1 μL drop of the solution. The recording channels were carefully coated 3–4 times (allowing drying between each coat) with the solution droplet. The sentinel channels were coated with a solution that did not contain GluOx (BSA-GA). MEAs were cured for at least 72 h prior to use. One day prior to recordings, all four Pt recording sites on the MEAs were electroplated with a size exclusion layer of mPD. Representative schematic of MEA coating can be found in **Figure 3A** (left).

### Instrumentation

Electrochemical preparation of the MEAs was performed using a multichannel Potentiostat (model VMP3). *In vitro* and *in vivo* measurements were conducted using a multichannel FAST16 mk-IV system (Fast Analytical Sensor Technology Mark IV, Quanteon, LLC, Nicholasville, KY) with reference electrodes consisting of a glass enclosed Ag/AgCl wire.

### *In vitro* MEA Calibration

On the day of amperometric recording, each MEA was calibrated *in vitro* to determine slope (nA/μM; sensitivity to glutamate), limit of detection (μM; lowest amount of glutamate reliably recorded), and selectivity (ratio of glutamate to ascorbic acid). For calibration, a constant potential (+0.7 V) was applied to the MEA against an Ag/AgCl reference in 40 ml of stirred 0.05 M PBS (pH 7.1–7.4; 37°C) in a beaker. After the current detected by the MEAs equilibrated to baseline (~20 min), 500 μL of ascorbic acid was added to the beaker to assess a readily oxidizable potential interferent that is in high concentration within the brain. This was followed by three subsequent additions of 40 μL of L-glutamate (20 μM) to confirm selectivity for glutamate and provide the slope of change in current as a function of changes in glutamate concentration. Last, 40 μL of H_2_O_2_ (8.8 μM) was also added to the beaker solution to test the sensitivity of the MEAs to the reporter molecule, peroxide. The final concentration consisted of (in μM): 250 ascorbic acid, 20, 40, and 60 glutamate, and 8.8 H_2_O_2_. In the present study, the average slope was 4.4 pA/μM, LOD 2.62 μM and selectivity 50.7–1. **Figure 3A** (right) depicts a representative MEA calibration.

### Microelectrode Array/Micropipette Assembly

For recordings in anesthetized rats, a glass micropipette was attached to the MEA for the local application of solutions. A single-barrel glass micropipette (1.0 × 0.58 mm^2^, 6″ A-M Systems, Inc., Sequim, WA) was pulled using a Kopf Pipette Puller (David Kopf Instruments, Tujunga, CA). Using a microscope with an eyepiece reticle, the pulled micropipette was bumped against a glass rod to have an inner diameter of 7–13 μm (10.5 μm ± 0.2). Clay was used to place the tip of the micropipette equidistant between the four Pt recording sites. The assembly was secured using Sticky Wax (Kerr Manufacturing Co.) and while the wax was still soft the micropipette was adjusted such that its tip was within 65 ± 6 μm from the surface of MEA. The assembly was allowed to cure for ~10–15 min and rechecked for distance before use in experiments.

### Surgery and Coordinates for Amperometric Recordings

Immediately after behavioral assessment, sham and FPI rats were anesthetized (1.5 g/kg urethane, i.p.). Following cessation of a toe pinch withdrawal reflex, each rat was then placed in a stereotaxic frame (David Kopf Instruments) with terminal ear bars. Body temperature was maintained at 37°C with Deltaphase^®^ isothermal pads (Braintree Scientific, Inc., Braintree, MA). A midline incision was made, and the skin, fascia, and temporal muscles were reflected to expose the skull. A bilateral craniectomy exposed the stereotaxic coordinates for the S1BF and VPM. Dura was then removed prior to the implantation of the MEA. Brain tissue was kept moist through the application of saline soaked cotton balls and gauze. Finally, using blunt dissection, a 200 μM diameter Ag/AgCl reference electrode was placed in a subcutaneous pocket site remote from the recording areas.

### *In vivo* Amperometric Recording

Amperometric recordings performed here were made similar to published methods (40, 42, 46). Solutions of either (in mM): KCl (120), NaCl (29), CaCl_2_ (2.5) in ddH_2_O, pH (7.2–7.5) or L-glutamate (in μM) [L-glutamate (100) in 0.9% sterile saline, pH 7.2–7.6] were filtered through a 0.20 μm sterile syringe filter (Sarstedt AG & Co., Numbrecht, Germany) and loaded into the affixed single-barrel glass micropipette using a 4-inch, 30- gauge stainless steel needle with a beveled tip (Popper and Son, Inc., NY). The open end of the single-barrel glass micropipette was connected to a Picospritzer III (Parker-Hannifin Corp., Mayfield Heights, OH). Solutions were locally applied from the glass micropipette with settings to dispense nanoliter (nL) quantities over a 1 s time period using the necessary pressure of nitrogen (inert) gas. The volume ejected was monitored using a stereomicroscope (Meiji Techno, San Jose, CA) fitted with a calibrated reticle. *In vivo* recordings were performed at an applied potential of +0.7 V compared to the Ag/AgCl reference electrode. All data were recorded at a frequency of 40 Hz, amplified by the headstage (2 pA/mV) without signal processing or filtering of the data. Glutamate and KCl-evoked measures were recorded in both hemispheres in a randomized and balanced experimental design to mitigate possible hemispheric variations or effect of anesthesia duration by investigators blinded to the injury status. The amperometric recordings were collected from multiple independent cohorts on consecutive days that contained both male and female rats from sham and FPI groups.

### Coordinates for Recordings

Using the Dual Precise Small Animal Stereotaxic Frame (Kopf, Model 962), the MEA assembly was slowly vertically lowered at 0.3 mm steps from the dorsal site. The MEA-micropipette assembly was lowered through the S1BF for males [from bregma (in mm): anteroposterior, ±2.3; mediolateral, ±5.0; dorsoventral, −1.1 to −2.1)] and females [from bregma (in mm): anteroposterior, ±2.2; mediolateral, ±5.0; dorsoventral, −0.8 to −2.0)]. The MEA-micropipette assembly was lowered through VPM for males [from bregma (in mm): anteroposterior, ±3.5; mediolateral, ±2.68; dorsoventral, −4.3 to −6.2)] and females [from bregma (in mm): anteroposterior, ±2.3; mediolateral, ±2.68; dorsoventral, −4.0 to −5.8)] (48).

### KCl-Evoked Overflow of Glutamate Analysis Parameters

Once the electrochemical signal had reached baseline, 120 mM KCl was locally applied to produce an evoked glutamate overflow. Additional ejections of KCl were completed at 2-min intervals. Criteria for analysis required that the peak represent the maximum amount of glutamate overflow within the surrounding neuronal tissue, this is confirmed by smaller peak amplitudes from consecutive KCl ejections. Primary outcome measure was peak amplitude (μM) taken as the absolute height of the recorded peak.

### Glutamate Clearance Analysis Parameter

**Figure 2:**
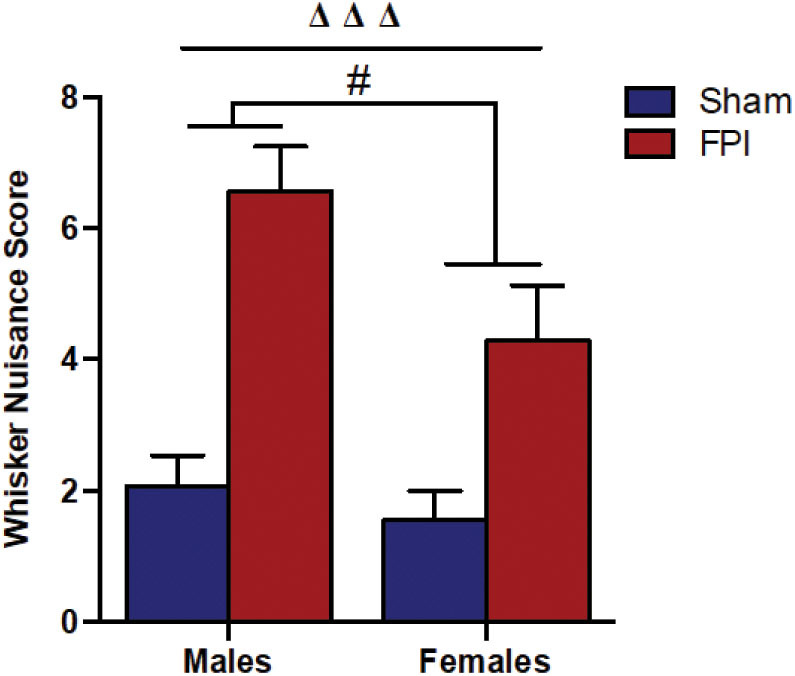
Comparison of whisker nuisance scores in WNT among male **(Left)** and female **(Right)** rats, subjected to either sham or midline fluid percussion injury (FPI). Data were collected during three 5-min time bins across a 15-min testing session in an open box. Two-way ANOVA [(injury: sham vs. FPI) and (sex: male vs. female)] where adjusted WNS (WNS + 1) was log-transformed to remove skewness. Sham: male, *N* = 12 rats; FPI: male, *N* = 17 rats; sham: female, *N* = 16 rats and FPI: female, *N* = 16 rats. Data expressed as mean ± SEM on original scale. ^ΔΔΔ^*P* < 0.001 compared to sham animals (overall FPI effect) and ^#^*P* < 0.05 compared to males (overall sex effect).

Once the baseline was reached and maintained for at least 10 min, 100 μM glutamate was locally applied into the extracellular space. Exogenous glutamate was released at 30 s intervals. In analysis, up to three peaks (with <10% variability) were selected based on a predetermined amplitude range of 8 to 18 μM to maintain similar Michaelis-Menten clearance kinetics. The parameters for the three peaks were then averaged to create a single representative value per recorded region per rat. Outcome measures analyzed included the uptake rate constant (k_−1_) measured as the first order decay rate of the glutamate signal (sec^−1^) and T_80_ duration (seconds) calculated as the time taken for 80% of the maximum amplitude of glutamate to clear the extracellular space. The uptake rate can be calculated using the uptake rate constant (k_−1_) multiplied by the peak's maximum amplitude, which normalizes for small variations (data not shown). We are presenting k_−1_ data that represent a similar outcome as the uptake rate due to amplitude matching. For a diagrammatic representation of these calculations, see Figure 2.

### MEA Placement Verification

Immediately following *in vivo* anesthetized recordings, rats were decapitated, brains post-fixed with 4% paraformaldehyde in PBS, and cryoprotected in serial solutions of 15 and 30% sucrose in Tris-buffered saline. Later, brains were sectioned at 40 μm to confirm MEA placement (see Figure S1).

### Statistical Analyses

Whisker nuisance score (WNS) is from 0 to 16. Electrochemical data were organized using Microsoft Excel (version 16.39). All outcome measures, unless otherwise stated, were averaged from multiple depths to create a single representative value for each rat prior to statistical analysis. Distribution of WNS and all electrochemical outcomes were evaluated for variability, and logarithmic transformation was applied to remove skewness for statistical methods. For log-transformation of the WNS, we adjusted the score by adding 1 to each reading since the lowest score was 0. Two-way ANOVA was used to compare each outcome measure between male and female rats and between FPI and sham groups. When there was evidence of an interaction between these two sets of factors, the FPI effect was reported separately for males and females; when there was no evidence of an interaction, FPI effect was reported for male and female rats combined, and sex difference was reported for FPI and sham rats combined. Linear mixed effects model was used to analyze data with multiple measurements from the same animal (e.g., electrochemical measurements at multiple depths). Spearman's rank correlation was employed for correlation analysis between WNS and evoked glutamate overflow or uptake rate constant (k_−1_) to assess whether the severity of hypersensitivity to the WNT was correlated with glutamate neurotransmission. This was performed separately for FPI male and FPI female rats. The estrous cycle for females was tracked daily for the entire duration of the study. Estrous cycle data are represented as percentage days spent in each phase over 28 days post-injury analyzed by two-way ANOVA followed by Fischer's comparisons. Significant changes in the time spent in each phase were *a posteriori* analyzed as a function of time (binned weekly) to determine whether the test was picking up acute or chronic changes. Graph Pad Prism (version 8.0) or R (version 3.5.3) were used to create graphs and perform statistical analyses. All tests were two-sided and *P*-levels < 0.05 were considered to be statistically significant for all tests except indicated. Data were graphically represented as mean ± standard error of the mean (SEM), regardless of any transformation performed in the statistical tests.

## Results

### Injury Characteristics

Injury characteristics including apnea, fencing response and righting reflex times were monitored in all rats immediately after injury as indicators of TBI severity. Apnea times were determined from injury to the return of spontaneous breathing. Righting reflex time was noted as the time of injury until return of an upright position. Baseline body weight for males was ~34% higher in comparison to female rats at the time of surgery. Due to differences in body weights, females received a marginally lower (6%) FPI impact with atmospheric pressure ranging from 1.9 to 2.1 for males and 1.8 to 2.0 for females. The weighted pendulum arm was adjusted for female sex to produce a righting reflex time of 5–9 min and survival rates of matched male rats based on previous publications (49, 50) and procedures previously established in our research program indicating less injury force in females. The average righting reflex times, similar among the brain-injured male and female rats, were 389.3 ± 17.08 and 411.0 ± 25.46 s, respectively (see Table S1).

### Sensory Hypersensitivity to Whisker Stimulation Among Males and Females After Diffuse TBI

It is understood that TBI impairs sensory processing contributing to behavioral morbidity (51–53). Previously, we reported late-onset post-TBI sensory hypersensitivity to whisker stimulation in male rats (40). The WNT serves as a useful test to measure late-onset sensory hypersensitivity associated with impaired sensory processing after brain injury (34). Two-way ANOVA showed that there was no evidence of interaction between injury and sex (*P* = 0.46), indicating that male and female rats shared a similar injury effect. In the absence of interaction effects, median adjusted WNS (WNS + 1) showed FPI rats had higher (127%) scores compared to sham rats [95% confidence interval (CI): 66.4–210%, *P* < 0.0001] and the overall scores were lower (31.8%) in females compared to males (sex effect) (95% CI: 7.0–50.0%, *P* = 0.019; Figure 2).

Vaginal cytology was used to track the four stages of the estrous cycle in all females. Table 1 presents a summary of the effects of sham and FPI on percentage of days female rats spent in each phase of estrous out of the 28 days post-injury (28 DPI). The two-way ANOVA revealed a significant injury × phase interaction (*P* = 0.012). The follow-up comparisons indicated that FPI females spent significantly more time in diestrus (*P* = 0.02) and less time in the estrus (*P* = 0.002). However, no significant differences were observed after FPI on number of days in proestrus and metestrus. An *a posteriori* assessment of diestrus and estrus over weeks post-injury in female rats was carried out to identify if the 28-day assessment was influenced by FPI (see Figures S2A, S2B). In the case of diestrus, a two-way ANOVA (week post-injury × injury; on log-transformed measurements) revealed a significant effect of injury (*P* = 0.008) with increased number of days in diestrus among FPI females, but not as a function of weeks (*P* = 0.452). There was a reduction in number of days in estrus as a function of weeks that approached significance as a function of injury (*P* = 0.053), but not as a function of weeks (*P* = 0.338). Thus, changes in the estrous cycle after FPI were not skewed by earlier time points. The key point is that FPI-induced chronic changes to the estrous cycle, similar to clinical observations (24) and capable of influencing long-term changes in circulating gonadal hormones.

**Table 1:** Percentage of days female rats spent in each phase of estrous over the 28 DPI for sham or midline fluid percussion injury (FPI).

**Estrous phase**	**Groups**	
	**Sham**	**FPI**
Proestrus	24.11 ± 2.24	22.54 ± 1.49
Diestrus	28.79 ± 1.20	34.59 ± 1.45^*^
Metestrus	12.29 ± 1.81	14.74 ± 1.49
Estrus	32.81 ± 2.57	24.99 ± 1.84^**^

*Values are mean ± SEM (N = 16/group). Data analyzed by two-way ANOVA followed by Fischer's LSD*.

* P < 0.05 and

** *P < 0.01 compared to same estrous phase sham*.

### Enhanced Evoked Glutamate Overflow Within VPM of Female Rats

**Figure 3:**
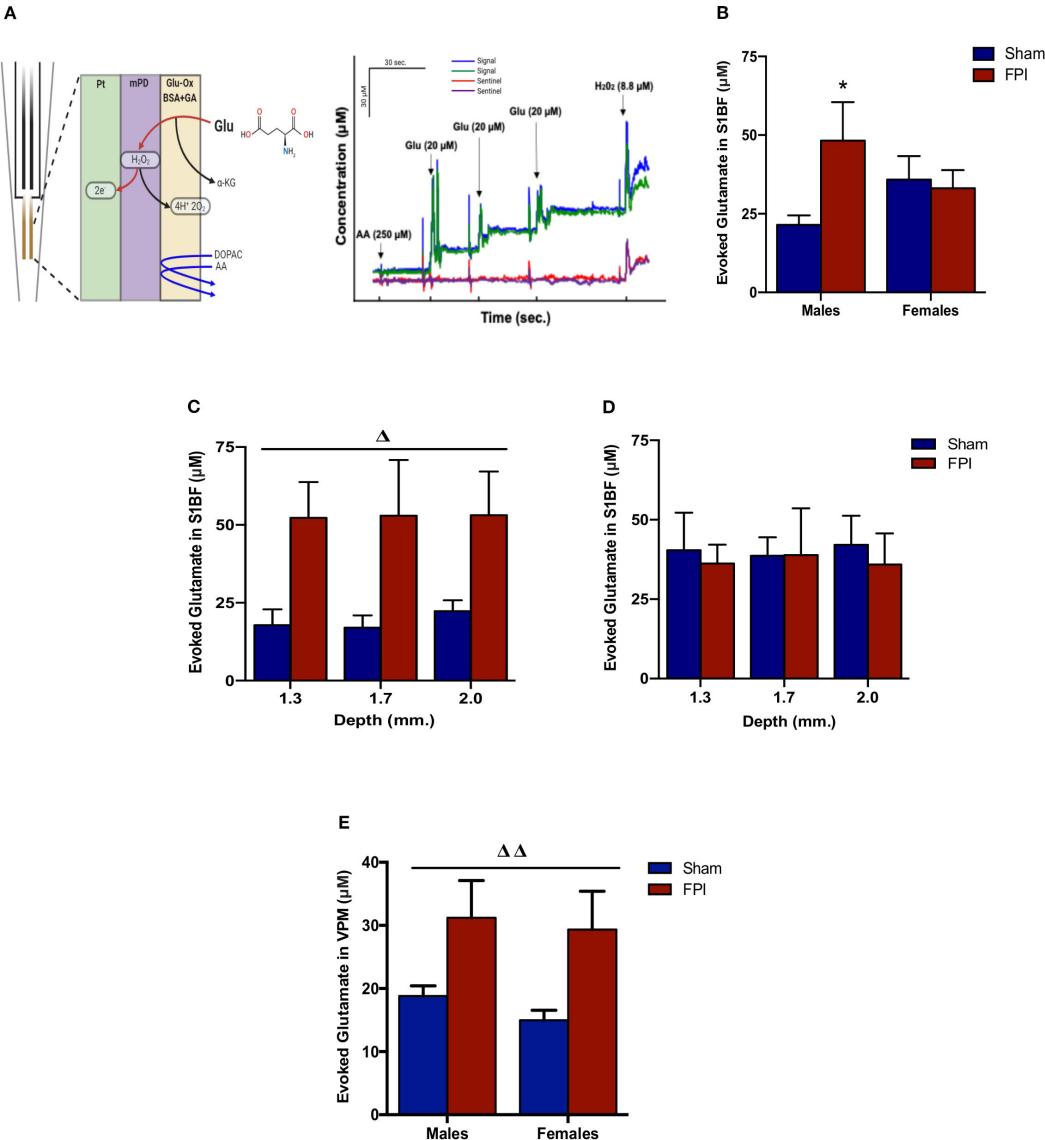
Evoked glutamate overflow in brain-injured circuitry among male and female rats subjected to either sham or midline fluid percussion injury (FPI). **(A)** Representative schematic of MEA coatings (left) and *in vitro* calibration (right). The ceramic-based MEA is equipped with four separate platinum (Pt) recording sites, where coatings are applied to the front sites (green and blue) to make the MEA selective to glutamate detection. In 3A (right), arrows represent aliquots of solution of either 250 μM ascorbic acid (AA), 20 μM glutamate (Glu), or 8.8 μM H_2_O_2_. **(B)** KCl-evoked glutamate overflow analyzed at 28 days post-injury by enzyme-based MEAs coupled with amperometry in the barrel fields of S1BF of male and female rats subjected to either sham or midline fluid percussion injury (FPI). **(C)** The depth profile of evoked glutamate overflow in the S1BF of male rats. Linear mixed effects model was applied to log-transformed measurements. **(D)** The depth profile of evoked glutamate overflow in the S1BF of female rats. **(E)** Evoked glutamate overflow in the VPM of male and female rats. Two-way ANOVA (injury and sex) applied to the log-transformed measurements to remove skewness. Data expressed as mean ± SEM on original scale. ^Δ^*P* < 0.05 and ^ΔΔ^*P* < 0.01 compared to sham rats (overall injury effect) and ^*^P < 0.05 compared to sex-matched sham. Figure 3A (left) created with BioRender.com.

Isotonic KCl solution (120 mM) was locally applied to depolarize the synaptic microenvironment to assess glutamate stores. As shown in Figure 3B, two-way ANOVA (on log-transformed measurements) showed marginally significant evidence of interaction between injury and sex (*P* = 0.091), so FPI effects were reported separately for males and females. The follow-up comparisons indicated that FPI significantly enhanced evoked glutamate overflow in S1BF among male rats (median 82.6% higher, 95% CI 6.8–212%, *P* = 0.034), whereas FPI had no effect in female rats (*P* = 0.84). While depth profile (dorsal-ventral axis) of the S1BF showed increased glutamate overflow in FPI males (median 132% higher, 95% CI 20–347%; *P* = 0.012) in comparison to sham males (Figure 3C), no such effect was observable in females (FPI females vs. sham females) (Figure 3D). In VPM, the two-way ANOVA revealed a significant main effect of injury (*P* = 0.005), such that FPI significantly increased evoked glutamate overflow in male and female rats when compared to sham (see Figure 3E).

### Slower Glutamate Clearance in Females Within S1BF and VPM

**Figure 4:**
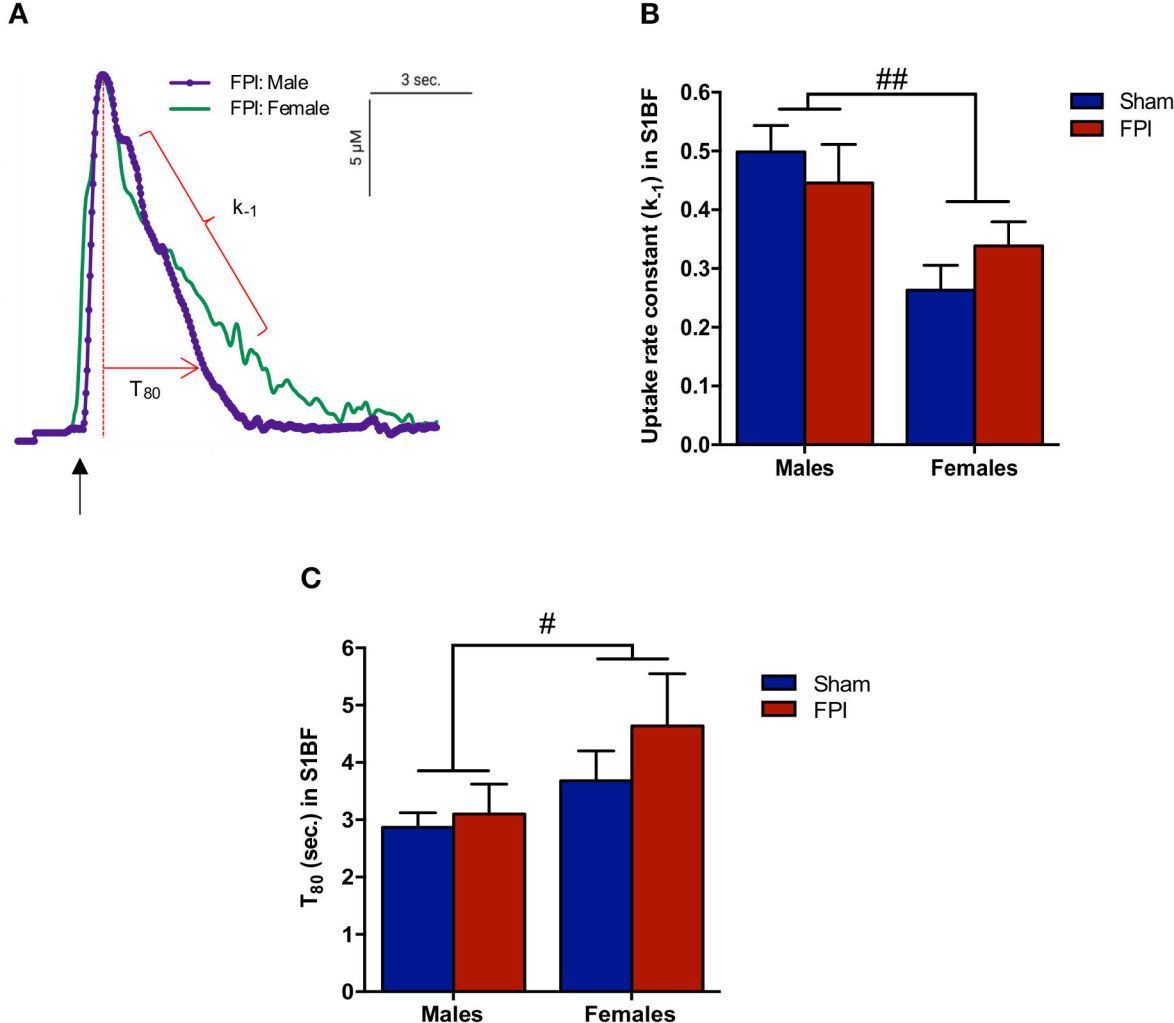
Extracellular glutamate clearance parameters among male and female rats subjected to either sham or midline fluid percussion injury (FPI). **(A)** Representative signals of extracellular glutamate clearance following local applications of glutamate through a micropipette in the S1BF (blue; males and green; females). An arrow represents application of 100 μM glutamate. The amplitude (μM) is calculated as the peak amplitude of the transient. Uptake rate constant (k_−1_) is calculated as the linear fit of the first order decay of the glutamate signal (s^−1^). T_80_ (sec.) is the time for the signal to decay 80% from the peak amplitude. **(B)** Extracellular glutamate uptake rate constant (k_−1_) in the S1BF of male and female rats subjected to either sham or midline fluid percussion injury (FPI). **(C)** T_80_ clearance time in S1BF of male and female rats at 28 days post-injury. Two-way ANOVA applied to the log-transformed measurements to remove skewness. Data represented as mean ± SEM. ^#^*P* < 0.05 and ^*##*^*P* < 0.01 compared to male rats.

**Figure 5:**
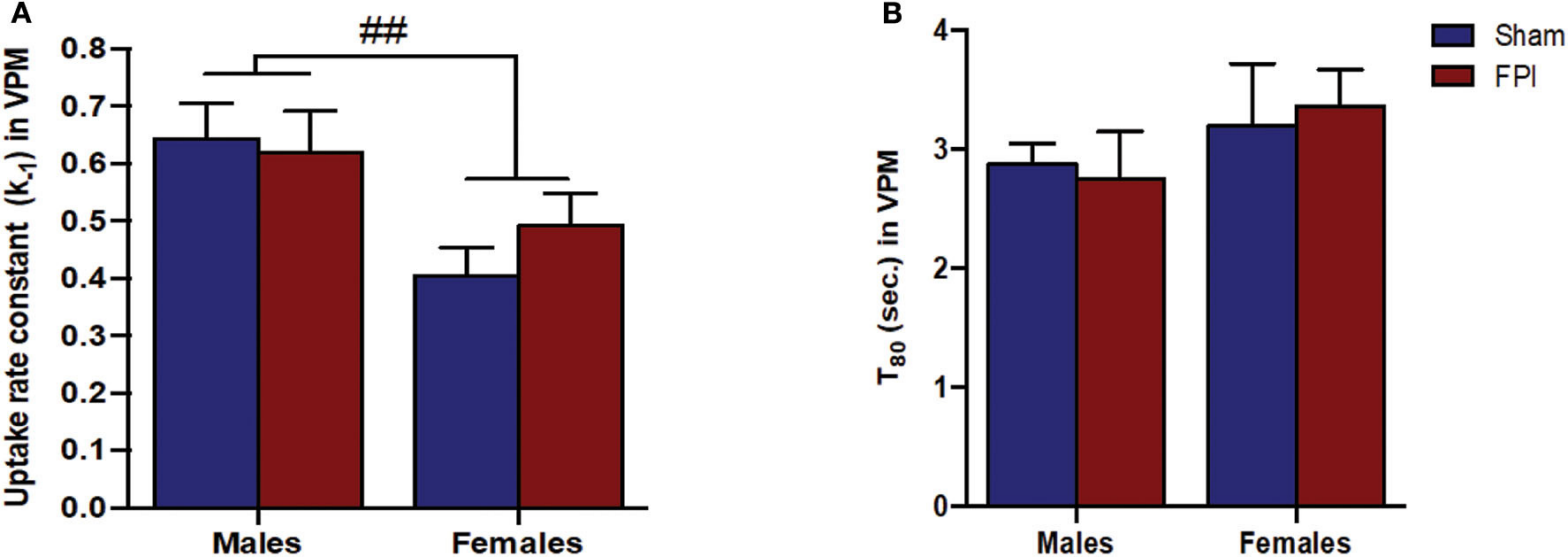
**(A)** The glutamate uptake rate constant (k_−1_) in the VPM of male and female rats subjected to either sham or midline fluid percussion injury (FPI). Data were analyzed by two-way ANOVA. **(B)** T_80_ clearance time (sec.) of glutamate in the VPM of male and female rats subjected to either sham or midline FPI. Two-way ANOVA applied to the log-transformed measurements to remove skewness. Data expressed as mean ± SEM. ^*##*^*P* < 0.01 compared to male rats.

Exogenous glutamate was locally applied to the extracellular space of the VPM and S1BF to evaluate glutamate clearance kinetics. Glutamate transporters regulate extracellular neurotransmitter levels based on Michaelis-Menten kinetics (54), so peaks were amplitude matched for analysis of glutamate clearance parameters. A sex-specific influence was observed with glutamate clearance within the thalamocortical circuit (representative signals in Figure 4A). For the glutamate uptake rate constant within the S1BF, there was no significant interaction between injury (sham vs. FPI) (*P* = 0.36), indicating that the uptake rate constant in males and females was not influenced by FPI. In contrast, as shown in Figure 4B, a significant sex effect was evident (*P* = 0.002), with females showing a lower glutamate uptake rate constant. As depicted in Figure 4C, while there was no evidence of a significant interaction between injury and sex for the T_80_ (*P* = 0.43), the T_80_ varied as a function of sex (*P* = 0.037), such that glutamate took longer to clear from the extracellular space in females in comparison to their male counterparts. However, there was no evidence of an FPI effect on T_80_ (*P* = 0.68). Likewise, the glutamate uptake rate constant in the VPM was also influenced by sex, with females having a lower uptake rate constant in comparison to males (*P* = 0.002; see Figure 5A), that was not altered by FPI (*P* = 0.36). Finally, there was neither FPI (*P* = 0.85) nor sex (*P* = 0.34) effect on the T_80_ (Figure 5B). These results are indicative of sex-dependent alterations in glutamate clearance as a function of sex, not injury. For additional validation, results of all electrochemical parameters were tested for rank order correlation with the corresponding WNS. While the increase in evoked overflow after TBI replicated previous experiments, the correlation between evoked glutamate overflow and severity of WNS was only significant (*P* = 0.01) for sham males in the S1BF. Further, no significant relationships were found in FPI animals (see Table S2).

## Discussion

Poor long-term outcomes after TBI are frequently associated with the number and severity of chronic post-TBI symptoms, with clinical data indicating that sex could influence such outcomes (55–57). Yet, studies focusing on post-traumatic circuit disruption and repair in naturally cycling females have received little attention. Our present findings support that differential manifestations of post-traumatic symptoms can be, in part, related to sex. We found TBI-induced sex-dependent changes in glutamate signaling, in terms of release and uptake profiles, associated with sensory hypersensitivity to whisker stimulation. Although the precise factors responsible for sex-dependent changes were not addressed in these experiments, they may be related, in part, to sex hormones. Additional studies are warranted to further investigate the role of specific hormones and receptor mediated events to draw more conclusive evidence in their control of neurotransmission and response to repair and compensation over time following diffuse brain injury. These results shed light on sex-dependent influences on glutamate neurotransmission and associated late-onset sensory hypersensitivity with and without TBI.

### Sensory Hypersensitivity and Estrous Cyclicity

Axons that survive the diffuse insult (adjacent to damaged axons) can impair circuit function creating maladaptive neuronal circuitry and activation, axonal damage, altered signaling cascades, and synaptic dysfunction (35, 58, 59). Impaired circuit function in the whisker barrel disrupts somatosensory processing through the whiskers of rats, a primary sensory modality, similar to vision in humans, and is implicated in the agitated response displayed during whisker stimulation (52, 60). In agreement with previous findings (34, 40), we found that TBI induced sensory hypersensitivity to whisker stimulation in both sexes, however, females displayed an overall lower magnitude of sensory hypersensitivity than males. The whisker barrel circuit is structurally well-organized with somatotopic arrangement that, while being a simple circuit, mediates complex behaviors (61). Our research and others demonstrate that late-onset sensory hypersensitivity after FPI parallels neuropathology, neuroinflammation, gliosis, circuit reorganization, changes in electrophysiological activity, and neurotransmission indicating a number of ongoing sequelae of events in the relays of the whisker barrel circuit leading up to the development of this aberrant behavioral phenotype (36, 38, 62–65). The sensory hypersensitivity to stimulation is indicative of a sensory gating defect after TBI that manifests as a defensive and apprehensive phenotype to tactile stimuli (66). Moreover, the apprehensive behaviors observed here after experimental TBI are similar to the hypersensitivity to visual stimuli, agitation, irritability, and hyperarousal reported in male and female TBI patients (39, 60, 67). Our results lend further credence to the whisker barrel circuit representing a useful somatosensory model to elucidate circuit disruption and repair that causes abnormalities in sensory processing after TBI (68), however, further pathological assessment in the whisker barrel circuit of female rats may highlight important sex differences.

TBI has been reported to cause hypothalamic-pituitary-gonadal (HPG) axis dysfunction and endocrinopathy that can impact the menstrual cycle in women (69–71). In these experiments, we evaluated the estrous cycle for 28 days after injury or sham surgery. Our data indicate that FPI females spend more time in the diestrus phase (low estrogen and high progesterone levels) and less time in the estrus phase over 28 days post-injury. When breaking this down to a week-by-week *a posteriori* analysis, the differences are a function of injury and not time, indicating that by 1 month post-injury normal cycling had not resumed (Figure S2). Previous TBI reports in clinical and rodent studies have also showed disruption of normal hormonal cyclicity (25, 29). Prolonged diestrus indicates longer secretion of progesterone with low levels of luteinizing hormone (LH) (72) that can interfere with ovulation. Additional clinical observations in women have reported abnormal menstrual pattern after TBI associated with amenorrhea (23) linked to variations in cortisol levels (73). Daily assessment of estrous cycle in these subjects was the optimal approach to link hormonal profiles of the cycle phases at any given time due to the repeated stress associated with blood draws that can downregulate neurotropic factors, including BDNF, that could mediate circuit repair after injury [reviewed by (74)]. Future studies directly evaluating the role of ovarian hormones in neurotransmission and neuroplasticity after TBI are necessary to further explain the sex-differences detected in these studies. Also, more detailed studies are required to test the influence of circulating hormones at the time of injury and at time of behavioral testing.

Age-matched naturally cycling females were used in these experiments to complement the history of FPI experiments using male Sprague-Dawley rats (300–350 g; 10–12 weeks old) with several studies of comparable behavioral, pathological, physiological, and molecular data available in males (40, 50, 75). At 10–12 weeks of age, rats are sexually mature, but have not reached social maturity, with literature estimating the translational relevance of this age to be late-adolescence/young adult (76, 77). In juvenile and aging TBI research, women and female rodents would not be actively cycling, thereby having lower circulating gonadal hormones levels, where post-menopausal women have been indicated to respond similar to males (78). However, an epidemiological study of TBI in pediatric patients indicates that endocrinopathies peak within 2 years of the initial TBI, were more prevalent in females, and were predominantly reported as precocious sexual development (79). In the geriatric population, non-survivors of TBI were more likely to be males (80). Together, these data indicate that sex-differences may be prevalent in all age groups after TBI, where severity and type of morbidities (and mortality) may change based on the sex and age at injury, warranting further investigation and inclusion of females cohorts across all age groups in translational studies. In fact, impaired circuit function can also contribute to gonadal hormone deficiency, recognized as an important consequence of TBI-induced hypothalamic-pituitary-gonadal (HPG) dysfunction and has translational implications for therapeutic strategies.

Females have several factors that can influence outcome measures, including (but not limited too) circulating gonadal hormones, thickness of skull, size of brain, smaller mean axonal diameter (similar to clinical reports), muscle mass, and metabolic processes (81–83). The presence of estrogen in females has been shown to be neuroprotective in most of the animal models of neurodegenerative diseases and genetic mechanisms that control for the sex differences may also influence the pathophysiology. For these experiments, in order to maintain similar righting reflex times (our primary inclusion factor) between males and females, the force of the injury was decreased, with the potential that the pathology is decreased. Another option is to hold the injury force constant with the potential to induce greater pathology. With the paucity of data available in sex differences, these sex-related factors should be considered in the context of the questions asked and approach toward the answers. At this time, inclusion of detailed methodology and transparency is necessary as we evaluate for specific mechanisms responsible for increasing reports of sex differences accumulating in the TBI literature.

### Evoked Glutamate Overflow in Brain Injured Circuitry and Behavioral Manifestations

Several studies have documented the sequelae of events associated with glutamate dysregulation after mild TBI [reviewed by (84)]. Our previous report indicated that TBI-induced hypersensitivity to whisker stimulation is correlated with alterations of thalamocortical glutamate activity (40) suggesting that the neural correlates of hypersensitivity to sensory stimuli can be associated with evoked neurotransmitter release. In males, we found evoked glutamate overflow was elevated in S1BF and VPM 1 month after TBI, similar to our previous reports (40), which parallels with damage and repair of corticothalamic projections over time post-injury (85). Further, our results showed that in females, elevated evoked glutamate overflow was restricted to the VPM, with no change in the S1BF. Extensive investigations on estrogen after ischemia reveal both neuroprotective and neurotoxic influences over neuroinflammation, apoptosis, growth factor regulation, vascular modulation, and excitotoxicity that could mediate differential outcomes in diffuse TBI [reviewed in (86, 87)]. More studies are needed to understand the role of sex hormones in influencing evoked-glutamate overflow.

Previous studies indicate that elevated evoked glutamate overflow after TBI in males was mediated presynaptically, and not due to changes in glutamate transporters at 28 DPI (40). We have previously reported changes in VPM neuron morphology over time, where there is an acute and subacute loss of processes followed by return of the number of processes by 28 DPI in male rats, that could contribute to changes in evoked glutamate overflow (38). There are several other mechanisms that could contribute to the loss of presynaptic homeostasis at 1 month post-injury that require further investigation. Elevated evoked-glutamate overflow in the VPM could be due to influence on the components of the neurotransmitter vesicle release machinery to increase glutamate release from the releasable pools of synaptic vesicles. Diffuse TBI has been shown to enhance synapse-specific complexin levels that function as a vesicle fusion clamp to regulate neurotransmitter release to enhance neuronal excitability (88). Hyperexcitability of dendrites has also been linked to altered expression of channel function and deafferentation (89, 90) suggesting that the sensory hypersensitivity and increased evoked glutamate overflow observed in the present study could arise from excessive circuit hyperexcitability after TBI. Further, cellular signaling pathways could also be chronically influenced, affecting glutamate release through synaptic changes in the glutamate receptors and/or voltage-gated calcium channels (84, 91). Moreover, presynaptic NMDA-type glutamate receptors (alpha amino- 3-hydroxy-5-methyl-4-isoxazole-propionic acid, AMPA) are also involved in neuroexcitatory activity that is initially activated by post-injury increases in glutamate that chronically augment neurotransmitter release in a feedback fashion (92, 93). Other factors that could play a role, but have not been investigated are; (i) anterograde microglial reactions to axonal injury (94) to release glutamate as a result of oxidative stress (95) or (ii) the dense white matter connectivity in the thalamus with glutamatergic bidirectional inputs from the S1BF and PrV that make the thalamus twice as vulnerable to DAI (96, 97). Primary inhibition to the whisker barrel circuit comes through the thalamic reticular nucleus, where controlled cortical impact injury has been indicated in the triggering of neuroinflammation and delayed reactive astrogliosis associated with the development of sleep disruption, indicating that GABAergic inhibition could be impaired to the whisker barrel circuit as well (98). These previous reports indicate a number of feasible approaches to further investigate the role of inhibitory: excitatory balance in the development of late-onset sensory hypersensitivity from whisker stimulation on the level of cells and circuits as well as a function of sex.

### Sex-Dependent Regulation of Glutamate Clearance in Brain-Injured Circuit

The lack of a TBI effect on glutamate clearance is consistent with our previous report (40). In addition, we presently observed a robust sex-difference, with female rats displaying lower glutamate k_−1_ in S1BF and VPM with increased T_80_ in S1BF, indicative of slower glutamate clearance in the female brain. However, T_80_ only increased in the S1BF, and not the VPM, indicating that while the rate of clearance is slower in females, the time glutamate spends in the extracellular space is the same in the VPM, supporting a change in *how* glutamate is being cleared from the extracellular space. The synaptic level and clearance of glutamate is primarily regulated by astrocytic sodium dependent excitatory amino acid transporters (EAATs) involving GLT1 and GLAST expressed by glia (99). We have previously reported that expression of glutamate transporters is not changed at 28 DPI in males within the whisker barrel circuit (40). An *ex vivo* radioactive uptake study reports that the female estrous cycle influences glutamate clearance (100). Although our data indicated TBI-induced changes in estrous cyclicity, there was no TBI effect on glutamate clearance, a differential effect involving *in vivo* assessment or possibly that the changes in estrous cycle were not robust enough to impact glutamate clearance. Given that glutamate homeostasis is tightly regulated under normal physiological conditions, the present results provide evidence that glutamate homeostasis may be differentially regulated between males and females. Sex differences have also been measured in glutamate receptor-mediated regulation of dopamine in rats, further supporting sexual dimorphism in the regulation of neurotransmission (101). There are also reports of estrogen receptor mediated inhibition of glutamate uptake activity (102, 103), where 17β-estradiol can increase protein levels of GLAST and GLT-1 and enhance glutamate clearance function [reviewed by (104)], which could normally play a role, in part, in how homeostasis is achieved. Another factor that warrants consideration is that the astrocytes express all estrogen receptor subtypes which provides multiple mechanistic pathways by which estrogen could mediate glutamate homeostasis, including upregulation of neuroprotective growth factors (105). In males, brain-injury induced astrocyte activation (indicated by GFAP staining) has been observed at 7 and 28 DPI in the VPM (38), indicating an active role for astrocytes in ongoing circuit reorganization and behavioral morbidity after TBI. Nevertheless, given that estrogen influences glutamate homeostasis, further exploration into its role in recovery following neurological insults is required. Ionic glutamate receptor perturbations have been associated with TBI, where sex differences in NMDA receptors on astrocytes can mediate regulation of glutamate neurotransmission and have a sexually dimorphic influence on behavioral and hormonal responses (106–108). Along these lines, we posit that sex-specific differences support the need for future studies assessing glutamate neurotransmission to power for females independently, or depending on outcome measures, power for increased variability in cohorts combining male and female animals.

## Conclusions

**Figure 6:**
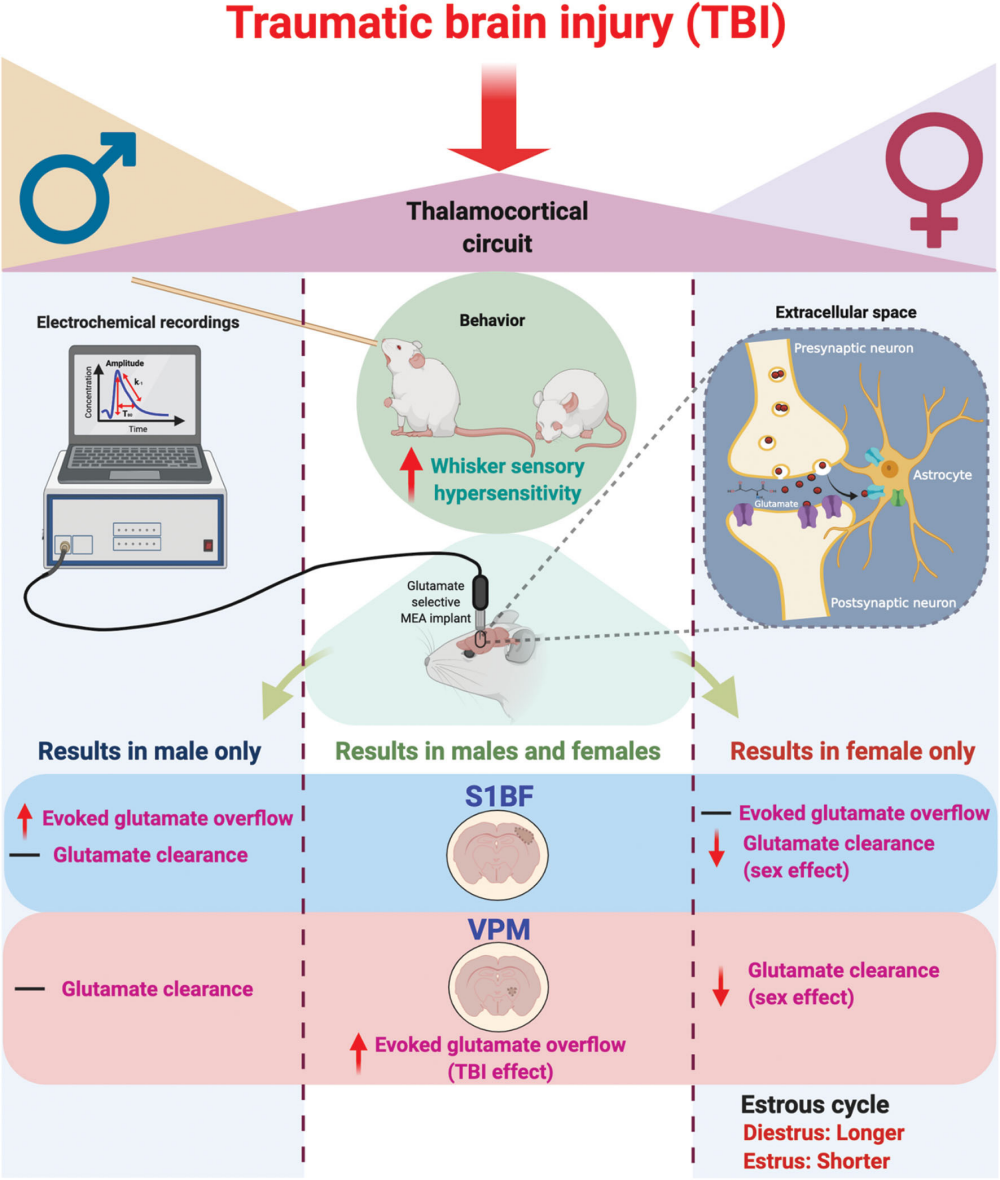
Schematic summary of chronic traumatic brain injury (TBI)-induced circuit disruption with sex-dependent late-onset sensory hypersensitivity and altered glutamate neurotransmission in whisker thalamocortical relays. In these experiments, we assessed the impact of diffuse TBI on changes in glutamate neurotransmission in the thalamocortical system that underlies the manifestation of late-onset sensory hypersensitivity to whisker stimulation in male **(Left)** and female **(Right)** rats. At 28 days post-injury (DPI), behavioral morbidity was detected as a function of injury in both males and females **(Center)**. Glutamate selective microelectrode arrays (MEAs) were used for electrochemical recordings within the extracellular space of primary somatosensory barrel field of the cortex (S1BF) and ventral posteromedial nucleus of the thalamus (VPM) relays of the whisker barrel circuit of anesthetized rats. In the S1BF, increased evoked glutamate overflow was observed only in male rats **(Left)**. In the VPM, there was an overall TBI effect on the depolarization-evoked overflow of glutamate in both sexes **(Center)**. Further, both in the S1BF and VPM, female rats had overall slower glutamate clearance parameters in comparison to males **(Right)**, with no injury effects detected. TBI females spent a significantly greater percentage of time in diestrus compared to shams, supported by an *a posteriori* analysis over weeks post-injury confirming increased time in diestrus. These data indicate irregular estrous cycles chronically after TBI. In summary, TBI induces sex-dependent post-TBI changes in somatosensory circuit function and long-term disruption of the estrous cycle (↑, increase; ↓, decrease; –, no change). Figure created with BioRender.com.

In these experiments, we tested the hypothesis that sex would not influence sensory hypersensitivity and associated *in vivo* amperometric extracellular recordings of glutamate neurotransmission within the behaviorally relevant thalamocortical circuit. Based on the findings from this study, we reject the hypothesis. Our results indicate that sensory hypersensitivity to whisker stimulation is present in both male and female rats at 1 month post-injury, however, overall scores were lower in females compared to males. Similar to previous results, evoked overflow of glutamate was elevated in the S1BF and VPM of males, yet this only occurred in the VPM of females. Also, similar to previous results, glutamate clearance was not impacted by injury at 28 DPI, however, there is a robust sex-difference indicating glutamate clearance in females is slower than in males. In addition, injured females had prolonged diestrus over the duration of 1 month post-injury in comparison to sham females (Figure 6), supporting clinical reports that TBI has a long-term impact on menstrual cycle. These results highlight the need to consider the effects of female hormonal status in TBI studies on the development of functional morbidity.

## Data Availability Statement

The raw data supporting the conclusions of this article will be made available by the authors, without undue reservation.

## Ethics Statement

This animal study was reviewed and approved by Institutional Animal Care and Use Committee Protocol (18–384) at the University of Arizona College of Medicine-Phoenix.

## Author Contributions

GK performed estrous cycle monitoring, amperometric analysis, analyzed the data, wrote the manuscript, and prepared the figures. CB performed estrous cycle monitoring, amperometric analysis and behavioral experiments. EC and EM assisted with amperometric experiments. CH performed statistical analyses and assisted with writing the results. PA and JL provided feedback on the manuscript. TT designed the research, assisted with experiments, analyzed the data, wrote the manuscript, and approved the final version. All authors approved the final version of the paper.

## Conflict of Interest

The authors declare that the research was conducted in the absence of any commercial or financial relationships that could be construed as a potential conflict of interest.

## Abbreviations

TBI: traumatic brain injury
S1BF: primary somatosensory (barrel) cortex
VPM: ventral posteromedial nucleus of the thalamus
MEA: microelectrode array
FPI: fluid percussion injury.
