# Supplementary material for: Traumatic Brain Injury-Induced Sex-Dependent Changes in Late-Onset Sensory Hypersensitivity and Glutamate Neurotransmission
Source: Front Neurol. 2020 Aug 5;11:749. doi: 10.3389/fneur.2020.00749 (PMC7419702; doi:10.3389/fneur.2020.00749)
Supplement: Supplementary file 1 [file Table_1.docx]

**Table S1.** Body weight and injury characteristics among brain-injured (FPI) male and female rats.

|  | Body weight (g) | ATM | Righting reflex |
| --- | --- | --- | --- |
| FPI-Males  (n =12) | 341.8 ± 7.7 | 2.13 ± 0.03 | 389.3 ± 17.0 |
| FPI-Females  (n =13) | 227.8 ± 4.8 | 2.01 ± 0.04 | 411.0 ± 25.4 |

FPI, fluid percussion injury
