# Supplementary material for: Traumatic Brain Injury-Induced Sex-Dependent Changes in Late-Onset Sensory Hypersensitivity and Glutamate Neurotransmission
Source: Front Neurol. 2020 Aug 5;11:749. doi: 10.3389/fneur.2020.00749 (PMC7419702; doi:10.3389/fneur.2020.00749)
Supplement: Supplementary file 2 [file Table_2.docx]

**Table S2.** Relationship between WNS and glutamate transmission (electrochemical measures) among brain-injured (FPI) male and female rats.

| Sensory hypersensitivity | Group | Electrochemical measures | rho (*P* value) | |
| --- | --- | --- | --- | --- |
|  |  |  | S1BF | VPM |
| WNS | Sham-Males | Evoked release | 0.820 (0.01) | -0.214 (0.66) |
|  |  | Uptake rate constant (k_-1_) | 0.622 (0.11) | 0.067 (0.86) |
|  | FPI-Males | Evoked release | 0.017 (0.96) | -0.175 (0.63) |
|  |  | Uptake rate constant (k_-1_) | 0.476 (0.12) | -0.164 (0.63) |
|  | Sham-Females | Evoked release | -0.168 (0.62) | 0.280 (0.40) |
|  |  | Uptake rate constant (k_-1_) | -0.278 (0.46) | -0.019 (0.97) |
|  | FPI-Females | Evoked release | 0.291 (0.36) | -0.305 (0.36) |
|  |  | Uptake rate constant (k_-1_) | 0.079 (0.84) | 0.077 (0.82) |

Correlations were performed using Spearman’s rank-order approach (rho).
