# Supplementary figures and images for: Traumatic Brain Injury-Induced Sex-Dependent Changes in Late-Onset Sensory Hypersensitivity and Glutamate Neurotransmission
Source: Front Neurol. 2020 Aug 5;11:749. doi: 10.3389/fneur.2020.00749 (PMC7419702; doi:10.3389/fneur.2020.00749)

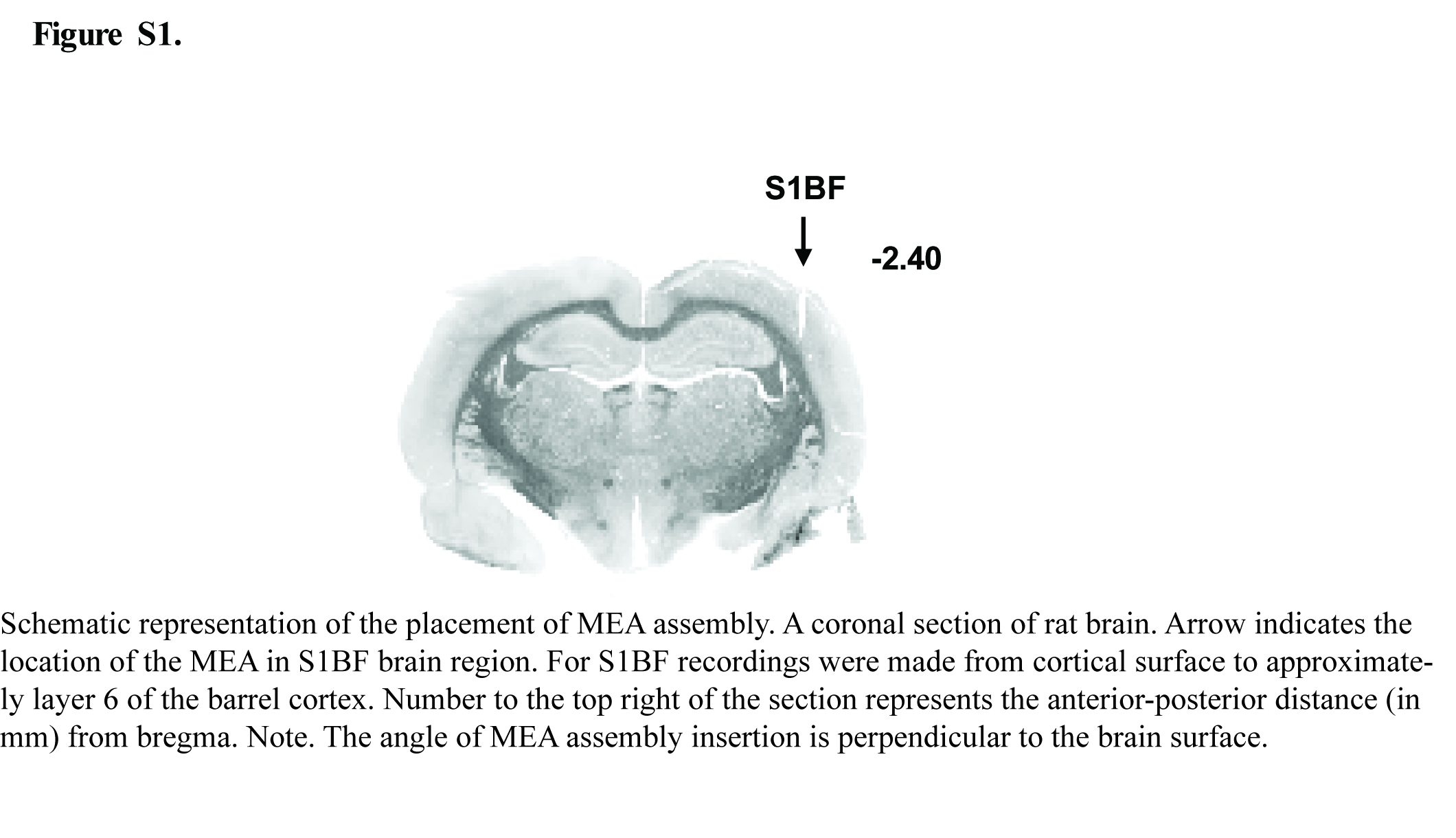

Supplement: Supplementary file 3 [file Image_1.TIF]

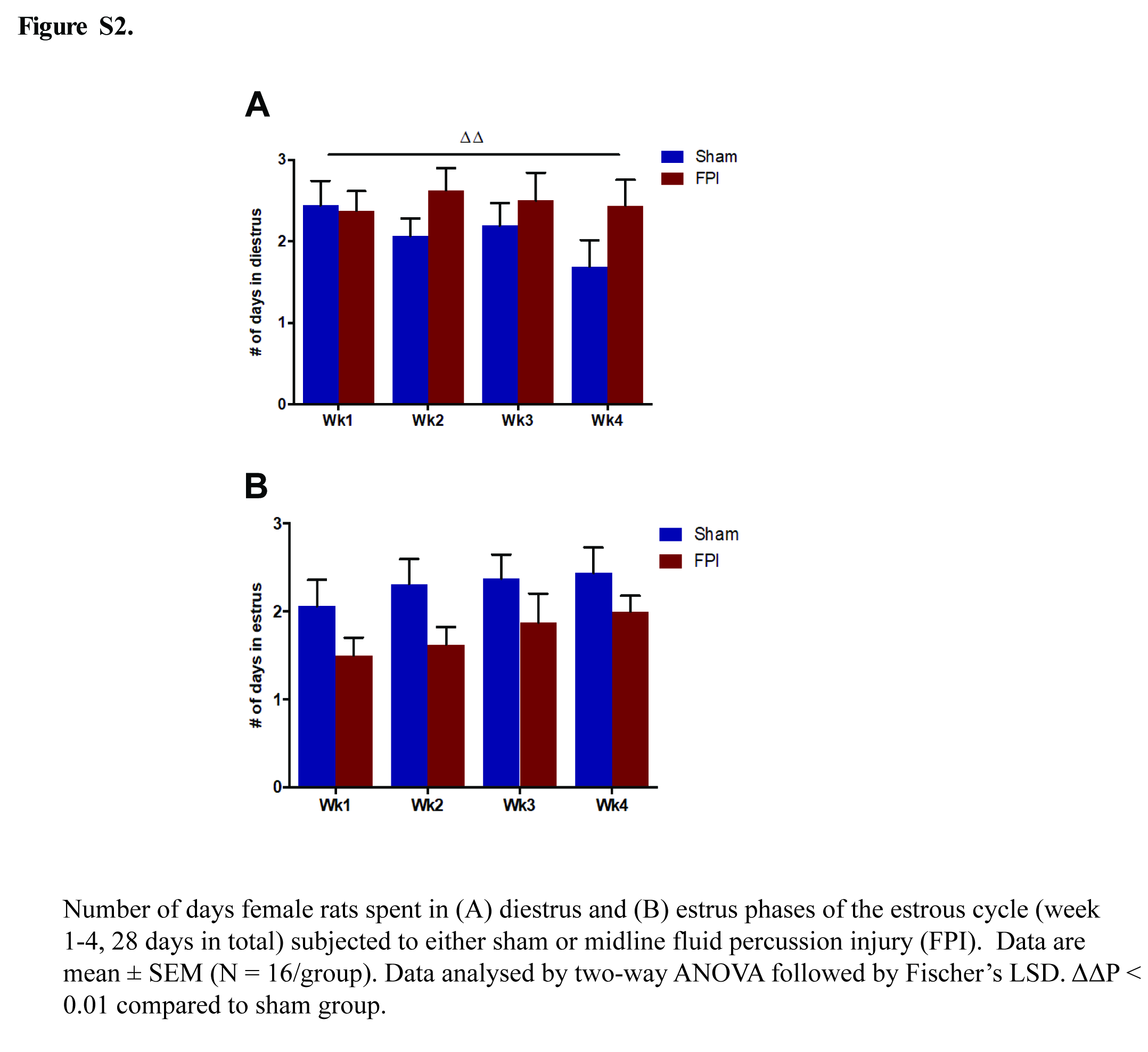

Supplement: Supplementary file 4 [file Image_2.TIF]
